# Urothelial cancer organoids: a tool for bladder cancer research

**DOI:** 10.1007/s00292-021-00988-9

**Published:** 2021-10-08

**Authors:** R. P. Meijer

**Affiliations:** University Medical Center Utrecht, Heidelberglaan 100, 3584CX Utrecht, The Netherlands

**Keywords:** Urothelial cancer, Organoids, Precision medicine, Drug resistance, Urothelkarzinom, Organoide, Präzisionsmedizin, Medikamentenresistenz

## Abstract

**Background:**

Bladder cancer ranks among the top ten most common tumor types worldwide and represents a growing healthcare problem, accounting for a large part of total healthcare costs. Chemotherapy is effective in a subset of patients, while causing severe side effects. Tumor pathogenesis and drug resistance mechanisms are largely unknown. Precision medicine is failing in bladder cancer, as bladder tumors are genetically and molecularly very heterogeneous. Currently, therapeutic decision-making depends on assessing a single fragment of surgically acquired tumor tissue.

**Objective:**

New preclinical model systems for bladder cancer are indispensable for developing therapeutic strategies tailored to individual patient and tumor characteristics. Organoids are small 3D tissue cultures that simulate small-size organs “in a dish” and tumoroids are a special type of cancer organoid (i.e., malignant tissue).

**Materials and methods:**

Since 2016, we have collaborated with the renowned Hubrecht Institute to provide proof of concept of tissue-based bladder tumoroids mimicking parental tumors. We have developed a living biobank containing bladder organoids and tumoroids grown from over 50 patient samples, which reflect crucial aspects of bladder cancer pathogenesis.

**Results:**

Histological and immunofluorescence analysis indicated that the heterogeneity and subclassification of tumoroids mimicked those of corresponding parental tumor samples. Thus, urothelial tumoroids mimic crucial aspects of bladder cancer pathogenesis.

**Conclusion:**

Research with urothelial tumoroids will open up new avenues for bladder cancer pathogenesis and drug-resistance research as well as for precision medicine approaches.

## Abstract (de)

**Hintergrund:**

Blasenkrebs gehört zu den 10 häufigsten Tumorarten weltweit und ist ein zunehmendes Problem, da es für einen großen Teil der Gesamtkosten des Gesundheitswesens verantwortlich ist. Chemotherapie wirkt bei einer Untergruppe von Patienten, verursacht aber schwere Nebenwirkungen. Tumorpathogenese und Mechanismen der Medikamentenresistenz sind weitgehend unbekannt. Präzisionsmedizin versagt beim Blasenkrebs, weil Blasentumoren genetisch und molekular sehr heterogen sind. Derzeit hängt die Therapieentscheidung von der Beurteilung eines einzigen Fragments von chirurgisch gewonnenem Tumorgewebe ab.

**Ziel:**

Neuartige Systeme präklinischer Modelle für Blasenkrebs sind unverzichtbar für die Entwicklung therapeutischer Ansätze, die auf den einzelnen Patienten und dessen Tumorcharakteristika zugeschnitten sind. Organoide sind kleine 3‑D-Gewebekulturen, die Miniorgane in einer Petri-Schale simulieren, und Tumoroide stellen einen speziellen Typ eines Krebsorganoids dar (d. h. malignes Gewebe).

**Material und Methoden:**

Seit 2016 kooperiert die Arbeitsgruppe des Autors mit dem angesehenen Hubrecht Institute (Utrecht, Niederlande) zur Entwicklung einer Proof-of-Concept-Studie zu gewebebasierten Blasentumoroiden mit Imitation des ursprünglichen Tumors. Dazu wurde eine lebende Biobank mit Blasenorganoiden und Tumoroiden aus mehr als 50 Patientenbiopsien angelegt, womit entscheidende Aspekte der Pathogenese von Blasenkrebs erkennbar werden.

**Ergebnisse:**

Histologische und Immunfluoreszenzuntersuchungen ergaben Hinweise darauf, dass die Heterogenität und Unterklassifizierung von Tumoroiden eine Imitation der entsprechenden Ergebnisse bei den ursprünglichen Tumorbiopsien darstellten. Folglich imitieren urotheliale Tumoroide entscheidende Aspekte der Pathogenese von Blasenkrebs.

**Schlussfolgerung:**

Die Forschung an urothelialen Tumoroiden wird sowohl neue Zugänge zur Pathogenese von Blasenkrebs und zur Erforschung der Medikamentenresistenz als auch zur Ansätzen in der Präzisionsmedizin eröffnen.

## Introduction

Bladder cancer ranks amongst the top five and top ten of the most common cancers in men and women, respectively [1]. Over 6500 patients were diagnosed with bladder cancer in the Netherlands in 2018. Bladder cancer patients are usually diagnosed through biopsies obtained by a cystoscope entering the bladder through the urethra. Approximately 73% of bladder cancer patients have non-muscle-invasive bladder cancer (NMIBC; Ta, CIS or T1), while the remaining 27% have muscle-invasive disease (MIBC; ≥ T2) or metastatic bladder cancer. Despite improved anatomical knowledge and refinement of surgical techniques, approximately 40–50% of non-metastatic muscle-invasive bladder cancers develop local relapse and/or metastatic disease, with a poor and unchanged outcome over the past 25 years (5-year survival of muscle-invasive bladder cancer, including metastatic disease, is approximately 35%) [6]. Multimodality treatment consisting of neoadjuvant cisplatin-based combination chemotherapy followed by radical cystectomy or radiation therapy has been shown to improve the outcome of this high-risk group of patients with muscle-invasive bladder cancer, albeit at best a 6.5% increase in overall survival at 5‑year follow-up [2, 7, 8]. One of the downsides of neoadjuvant chemotherapy is the uncertainty of chemosensitivity. Pathological complete response rates vary around 25%, meaning that three out of four patients will not therapeutically benefit from this otherwise highly toxic treatment [2]. Thus, evaluation of tumor biology and assessment of chemosensitivity of the individual bladder tumor is needed to guide personalized bladder cancer treatment [9, 10].

## Pathogenesis of bladder cancer

Several potential pathogenetic pathways have been described based on histopathologic and molecular observations. These include, for example, a pathway with hyperplasia (*FGFR3* mutation) and papillary Ta low-grade tumors (*PIK3CA* and *Stag*2 mutations) versus a pathway with dysplasia (*TP53* mutation), carcinoma in situ (*RB1* loss), and invasive/metastatic carcinoma (*ERBB2, ARID1A*, and *PTEN* mutations) [11]. Potential human bladder cancer stem cells/tumor-initiating cells have been isolated, showing features of basal cells residing at the tumor–stromal interface [12, 13]. Others have presented evidence for “non-basal” tumor-initiating cells in “more-differentiated” bladder cancers. Marker combinations corresponding to different urothelial differentiation states could stratify bladder cancer into clinically relevant subgroups, and tumors with the “least-differentiated” (basal) tumor-initiating cells had the worst outcome [14].

Recently, targeted sequencing has led to the identification of DNA mutations (genes such as *ERCC2/ERBB2* and varying sets of DNA damage response genes) that may predict chemosensitivity in bladder cancer [9]. Furthermore, molecular profiling studies have stratified bladder cancer into six different molecular subtypes: luminal papillary (24%), luminal non-specified (8%), luminal unstable (15%), stroma-rich (15%), basal/squamous (35%), and neuroendocrine like (3%). These molecular subtypes differ regarding underlying oncogenic mechanisms, infiltration by immune and stromal cells, and histologic and clinical characteristics [15]. Based on these studies, selection of patients by means of subtype-identifying biomarkers may enrich for responsiveness for either targeted, chemo-, or immunotherapy. The majority of current biomarker approaches rely on collection of tumor tissue. Liquid biopsies represent a promising new noninvasive approach. A rapid evolution in DNA profiling techniques has enabled detection of genomic aberrations in circulating cell-free tumor DNA (ctDNA) in peripheral blood. Depending on ctDNA fraction and the genomic regions of interest, various methods can be used for ctDNA analysis, including digital droplet PCR (ddPCR), ultra-deep sequencing with unique molecular identifiers, targeted next-generation sequencing, whole-exome sequencing (WES), or whole-genome sequencing (WGS). The broader next-generation sequencing panels allow more exploratory analyses of the genomic landscape. However, at present, this approach only appears feasible in patients with high ctDNA fractions, such as those with advanced metastatic disease. In patients with localized disease, including NMIBC, specific mutations can be detected using ddPCR [16].

Current pathogenicity profiles and derived biomarkers are insufficient to guide therapy installation. Notwithstanding the merits of these genetic findings, their clinical relevance is uncertain, given the highly heterogeneous genetic and molecular nature of these cancers. To add to the problem, there is an important difference between genetic tests as biomarkers for drug selection (indirect method and assumption of benefit) versus direct sensitivity testing (e.g., organoid testing). It is questionable whether the molecular subtype of a tumor is a stable “tumor-specific” factor. Several bio-informatics studies have shown that, for example, membership in the subtype TCGA cluster II/p53-like/infiltrated, is relatively unstable, and that luminal tumors may turn to the p53-like subtype after exposure to neoadjuvant chemotherapy [17]. These important alterations in tumor characteristics induced by drug exposure emphasize the relevance of longitudinal follow-up assessments for monitoring of drug response and drug resistance development.

Currently, few model systems exist that faithfully recapitulate the biology of the normal urothelium and bladder cancer. Cultures of primary mouse and human bladder cells have been reported but are limited due to their short lifespan [18]. The development of urothelial cells from induced pluripotent stem cells is one way to overcome this short lifespan [19]. Models for the study of bladder cancer include bladder cancer cell lines. These, however, fail to recapitulate many aspects of the original tumor and are often difficult to establish [20]. Genetic mouse models and orthotopic xenografts for bladder cancer have been created and studied [21]. These models are a faithful representation of the clinical manifestation but are time consuming to establish and maintain. Three-dimensional cultures of primary bladder cancer cells have recently been published [22]. This inspired us to apply our previously published organoid culture method of colorectal, pancreatic, and prostate cancer cells on bladder cancer [23]. Unlike the previously published 3D culture methods, organoids can be passaged multiple times and thereby massively expanded.

## Bladder tumoroids (cancer organoids)

Organoids are classified as “stem cell-containing self-organizing structures” that can be propagated for prolonged periods of time, and tumoroids are a special type of cancer organoid [4]. To enable growth of bladder (cancer) cells, we amended the original organoid protocol that was tailored to colorectal cancer [24]. Molecular signals that regulate renewal of the urothelium under physiologic conditions are incompletely understood. Upon bacterial infection, rapid proliferation of the urothelium is observed [25]. For this reason, we screened several culture medium conditions in which we included growth factors and inhibitors that were previously reported to influence urothelium culture [26]. In contrast to most published medium compositions for culture of either normal mouse urothelium or human bladder cancer, our bladder (cancer) organoid medium is completely defined and devoid of any animal products. In pilot literature, Shen et al. performed an analysis of 22 patient-derived bladder cancer organoid lines and demonstrated that mutational, molecular, and histopathologic profiles were highly concordant with the original “parental” tumors [27]. In our previous bladder organoid project, we prospectively collected samples from 53 bladder cancer patients (42 cystectomies and 11 transurethral resections; TURs) and processed these into organoid cultures [5]. We started 77 organoid lines from the 133 tissue samples we collected from 53 bladder cancer patients. In most cystectomy cases, we started cultures of both normal and tumor tissue, and in case of large tumors, we established several lines of different parts of the tumor. So far, we have managed to culture several organoid lines for more than 30 passages [5]. We have been able to generate several independent organoid lines from individual tumors which offer the potential to study intra-tumor heterogeneity in great detail. Upon analysis, we found that several tissues that were pathologically scored healthy were in fact premalignant (based on p53 status and karyotype). This confirms earlier findings that describe the “field effect” in bladder cancer: premalignant cells spread through the epithelium and, upon acquiring more mutations, give rise to the formation of bladder cancer [28].

Bladder cancer is a very heterogeneous disease at the molecular and genetic level [3]. Histologic and immunofluorescence analysis of the bladder cancer organoids (tumoroids) in our biobank show a large variation between organoids from different tumors [5]. The fact that bladder organoids can be grown for prolonged periods allows us to perform functional studies. For instance, we have seen that cells with a very common *FGFR3* mutation can proliferate for prolonged periods in the absence of any growth factor in the medium. This also opens the possibility to screen (novel) drugs on bladder tumoroids, potentially yielding urgently needed new therapeutics for the treatment of this disease. We randomly selected three organoid lines and exposed them to several commonly used chemotherapeutic agents. Organoids were subjected to a range of drug concentrations and incubated for 5 days. We observed differences when comparing various lines in their response to drug treatment. These experiments show how human bladder cancer organoids can be employed to determine the response to anti-cancer drugs. Potential applications include the screening of novel drugs and predicting tumor response to current treatment options. This will enable personalized medicine for bladder cancer patients. By evaluating chemosensitivity prior to treatment and identifying the patients who will likely respond to chemotherapy, we will be able to avoid toxicity and delay of definitive treatment in non-responders. However, it should be noted that drug testing in bladder tumoroids has (until now) never been validated with prospective bladder cancer patient outcome.

Bladder tumoroids provide a culture system in which genetic editing experiments can be performed to assess the genetic basis of drug response and resistance development. In the previous bladder organoid project, genetic editing experiments were performed. With CRISPR/Cas9 technology, we created knockout basal urothelium organoids. Basal cells (Ck5+) have been reported to be the cell of origin of muscle-invasive bladder cancer and carcinoma in situ. We successfully genetically targeted one very well-established tumor suppressor (*Trp53*) and one recently identified tumor suppressor in urothelial carcinomas (*Stag2*) in murine basal bladder organoids with Cas9 and gRNAs (guide RNAs) targeting the *Trp53* and *Stag2* genes. The *Stag2* gene is located on chromosome X, which creates the opportunity to generate knockouts in male cells with CRISPR/Cas9 with relative ease. We performed CRISPR/Cas9 genome editing for the *Trp53* and *Stag2* genes in organoids derived from male and female animals. We selected for cells that had inactivated their *Trp53* gene by adding an MDM2 inhibitor (nutlin) to the culture medium. Organoids derived from male and female animals were treated with *TP53* gRNAs and proliferated in the presence of nutlin. Single organoids were picked and expanded, and the targeted genomic locus was sequenced. In all clones tested, both alleles of the p53 gene were found to be mutated and, in addition, both alleles of the *Stag2 *gene had incurred a mutation (in female mice), showing that genomic editing in bladder organoids is feasible [5].

## Prospective population-based bladder cancer cohort infrastructure (ProBCI)

A comprehensive understanding of key molecular alterations in muscle-invasive and metastatic bladder cancer has resulted in the recent FDA approval of several new targeted and immune-oncologic drugs for patients in the metastatic setting. It is anticipated that in the near future, novel targeted and combination therapies will also be introduced for patients with localized disease, including high-risk non-muscle-invasive bladder cancer (carcinoma in situ and T1 bladder cancer). Although prospective randomized controlled clinical trials will remain the backbone for drug approval and implementation, rapid validation of these trial results in a real-world patient population is of vital importance. International trial results may differ in a more homogenous (Dutch) population, due to differences at the patient (demographic patient characteristics, genetic background) and the tumor level (differences in tumor biology/mutational or epigenetic regulation).

**Fig. 1:**
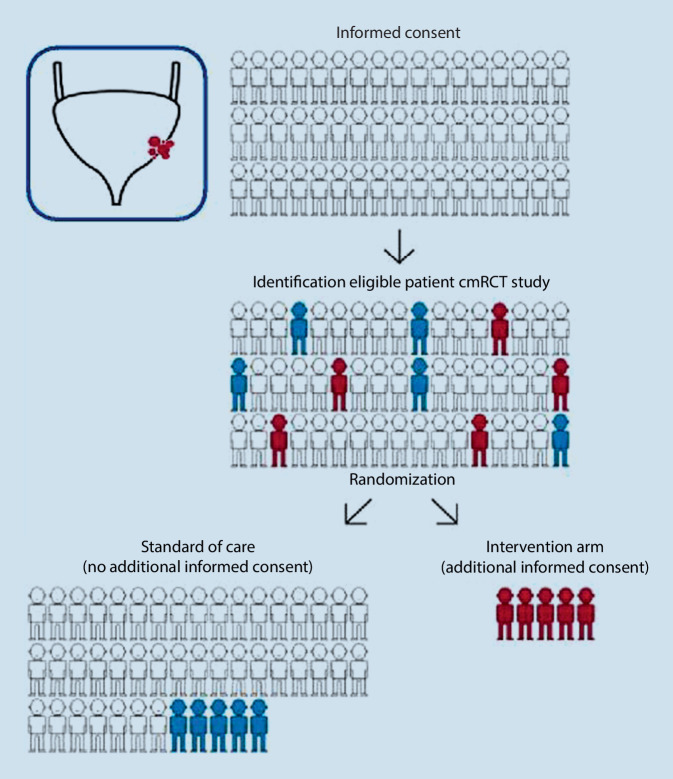
Prospective population-based bladder cancer cohort infrastructure (ProBCI), the “cohort multiple randomized controlled trial” design [29, 30]. An observational cohort of bladder cancer patients with the condition of interest is recruited and their outcomes regularly measured. Then, for each randomized controlled trial, information from the cohort is used to identify all eligible patients. Some eligible patients are randomly selected and form the intervention arm. The outcomes of these randomly selected patients are then compared with the outcomes of eligible patients not randomly selected, i.e., those receiving standard of care. This process can be repeated for further randomized controlled trials

Timely availability of relevant clinical data and biomaterials of patients diagnosed with high-risk bladder cancer and metastatic disease is crucial to enable first-time-right treatment. This necessitates an infrastructure aimed at identifying newly diagnosed bladder cancer patients, with available data on relevant patient and tumor characteristics, and, if applicable, data on already performed diagnostic and therapeutic procedures and outcomes. Such an infrastructure will facilitate the selection of eligible patients for specific intervention studies and thereby improve patient accrual. In addition, a random selection of patients who receive standard care can serve as a “control group” for single-arm (phase 2) trials. Also, results from clinical trials and prognostic and predictive biomarkers can be validated in patients treated within daily practice. Recently, the prospective population-based bladder cancer cohort infrastructure (ProBCI) was initiated in the Netherlands, which will be invaluable for building a prospective cohort of bladder cancer patients with “real-life” patient data and for collection of biomaterial and bladder tumoroids [29]. This research infrastructure is based on the “cohort multiple randomized controlled trial” design (cmRCT; Fig. 1; [30]). Such an infrastructure was also started successfully for colorectal cancer in the Netherlands in 2015 (www.plcrc.nl). The bladder cancer infrastructure will offer a real-world control group for innovative drug trials. Moreover, the clinical data and biomaterial collected within the infrastructure offer the opportunity to select patients with specific characteristics for trials and thus facilitate and improve trial accrual (Fig. 1). This nationwide infrastructure is aimed at quick and easy identification of eligible bladder cancer patients for new studies by using clinical data, biomaterials, and health-related quality of life (HRQoL) data of patients with high-risk non-muscle-invasive bladder cancer, muscle-invasive bladder cancer, and metastatic disease. This prospective cohort of bladder cancer patients will be used for clinical trials and biomarker validation to answer current unmet clinical needs in optimizing treatment strategies for high-risk muscle-invasive bladder cancer and patients who relapse or develop metastatic disease. Such an infrastructure based on the cohort multiple randomized controlled trial design is unique for bladder cancer worldwide and will result in robust “real-life” patient data in the rapidly evolving field of bladder cancer [29, 30]. This infrastructure is imbedded in the logistic framework of the Netherlands Cancer Registry (NCR) held by the Netherlands Comprehensive Cancer Organization (IKNL). Clinical data-collection has started in November 2017.

## Conclusion

The combination of the living bladder tumoroid biobank and the prospective bladder cancer cohort holds the potential to open up a new era in the field of bladder cancer precision treatment and to guide the development and application of new targeted drugs in daily clinical practice.

## Declarations

The investigations were carried out according to the specifications of the Central Ethics Committee of the Federal Medical Council.

The supplement containing this article is not sponsored by industry
